# Flexible circumferential bioelectronics to enable 360-degree recording and stimulation of the spinal cord

**DOI:** 10.1126/sciadv.adl1230

**Published:** 2024-05-08

**Authors:** Ben J. Woodington, Jiang Lei, Alejandro Carnicer-Lombarte, Amparo Güemes-González, Tobias E. Naegele, Sam Hilton, Salim El-Hadwe, Rikin A. Trivedi, George G. Malliaras, Damiano G. Barone

**Affiliations:** ^1^ Electrical Engineering Division, Department of Engineering, University of Cambridge, Cambridge, UK.; ^2^ Department of Clinical Neurosciences, University of Cambridge, Cambridge, UK.; ^3^ Division of Neurosurgery, Addenbrookes Hospital, Hills Road, Cambridge, UK.

## Abstract

The spinal cord is crucial for transmitting motor and sensory information between the brain and peripheral systems. Spinal cord injuries can lead to severe consequences, including paralysis and autonomic dysfunction. We introduce thin-film, flexible electronics for circumferential interfacing with the spinal cord. This method enables simultaneous recording and stimulation of dorsal, lateral, and ventral tracts with a single device. Our findings include successful motor and sensory signal capture and elicitation in anesthetized rats, a proof-of-concept closed-loop system for bridging complete spinal cord injuries, and device safety verification in freely moving rodents. Moreover, we demonstrate potential for human application through a cadaver model. This method sees a clear route to the clinic by using materials and surgical practices that mitigate risk during implantation and preserve cord integrity.

## Teaser

Flexible and thin circumferential neural interface allows stimulation and recording of the spinal cord.

## INTRODUCTION

The spinal cord, a cardinal constituent of the nervous system, functions as a pivotal conduit connecting the brain and the body. Any damage inflicted upon this complex structure invariably results in profound disability, marked by an irreversible loss of sensory, motor, and autonomic functions. Conversely, the capacity to effectively interface with the spinal cord presents a unique opportunity to unlock unprecedented potential in modulating and potentially restoring these crucial functions. This capability could markedly alter the landscape of therapeutic interventions for spinal cord injuries and associated neurological disorders (*1*). Recent advances in targeted spinal cord stimulation (SCS) have enabled the restoration of assisted walking in patients paralyzed by spinal cord injury (SCI) (*2*–*4*). These pilot studies rely on the open-loop initiation of a stimulation algorithm or external, closed-loop triggering via inertial measurement units. Alternatively, closed-loop SCS has also been used by decoding internal neural signals from the brain cortex (*5*, *6*). These approaches aim to restore motor function after SCI and focus on dorsal stimulation of the spinal cord by using commercially available SCS devices, which are primarily designed for the treatment of chronic pain. The mechanism has been postulated to be indirect activation of motor neurons through proprioceptive circuits located dorsally (*7*).

It has been demonstrated that direct stimulation of anterior motor neurons via ventral stimulation is more efficient in muscle activation and allows a greater degree of selectivity (*8*). This finding has led to recent efforts to develop ventral SCS devices (*9*, *10*). Furthermore, the spinal cord proximal to a site of injury, rather than external algorithms or brain cortex, represents a more viable and patient-friendly target for neural recording and decoding of motor intent (*11*, *12*). However, challenges exist for both the anatomical approach to the ventral spinal cord and the use of materials that limit iatrogenic neural injury. Furthermore, to date, spinal cord signals have yet to be used as control signals for closed-loop SCS due to limitations in recording and stimulation techniques, which only allow coverage of limited areas of the spinal cord. By harnessing advances in thin-film bioelectronic devices that more closely match the mechanical stiffness of neural tissue and conform to the spinal cord unique anatomy (*13*, *14*), we have designed a nonpenetrating conformal bioelectronic array capable of interfacing with the spinal cord circumferentially without causing iatrogenic SCI in rats. We have demonstrated how this approach allows comprehensive recording of neural signals around the spinal cord with high spatial and temporal dimensionality opening the door to whole cord signal analysis and simultaneous descending and ascending tract measurements. We combined recording and stimulation, and, in this proof-of-concept study, used these circumferential spinal cord implants (i360) to enable direct, low-latency spinal cord electronic bypass to restore hindlimb motion in acute SCI in rat models and tested their translation in human cadaveric spine models.

## RESULTS

### Device and surgical design

**Fig. 1.**
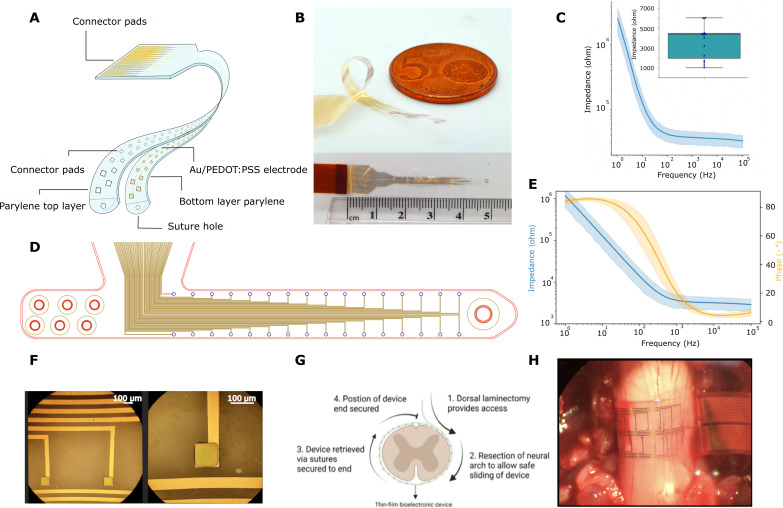
Device and surgical design. (**A**) Exploded illustration of the microfabricated I360 device design; the full fabrication of this device is detailed in fig. S1. (**B**) The I360 device shows looped flexibility and is presented next to a ruler. (**C**) Electrical impedance spectroscopy measurements from the I360 device *n* = 13 electrodes from two devices, insert is the range of impedance values seen at 1 kHz, a Bode plot is presented in fig. S2. (**D**) Electrodes outlay in the i360 device. (**E**) A representative Bode plot for a single i360 electrode, derived from frequency response analysis. (**F**) Optical image of the device electrodes, showing the PEDOT:PSS coating. (**G**) Process flow explanation of the surgical steps and device placement steps which were performed before recording or stimulation experiments. (**H**) An intraoperative image of the device wrapped around the longitudinal axis of the spinal cord. The bottom of the image shows the L1 superior facet. The device is shown wrapped around the spinal cord in the position where the i360 interface is used. In these experiments, the device is lying on the cord held only by capillary forces and applies no force or restriction.

To enable circumferential implantation around the spinal cord, we have designed a thin, flexible electrode array of 32 electrodes arranged in a staggered, linear configuration. Such a device maximizes the circumferential distribution of the electrodes distributed around the spinal cord while minimizing cross-talk contamination (*15*). The device concept, as well as optical images, is shown in Fig. 1 (A, B, and D). The parylene-C–based array with titanium and gold electronics was fabricated using photolithography techniques (*16*). Conducting polymer poly(3,4-ethylene) dioxythiophene (PEDOT) with poly(styrene sulfonate) (PSS) acting as counterion to PEDOT was used to decrease the impedance of the electrodes and thus enhance performance in both recording and stimulation (*17*). The low thickness of the parylene-C devices (4 μm) renders them highly flexible (*18*) allowing for minimal disturbance of movements of the spinal cord. The footprint of the electrode interconnect was minimized to allow the device to be implanted through the interval between the exiting nerve roots. The circumferential spinal cord interface devices (i360) were available in varying dimensions (8.90, 9.90, and 10.90 mm in lengths) to allow the experimenter to adapt to variations in rodent spinal cord anatomy and to implant the devices that maximized electrode coverage of the spinal cord circumference (*19*, *20*). The devices were characterized in vitro using electrical impedance spectroscopy (Fig. 1C) with individual device electrodes demonstrating low impedance (1 to 6 kilohm) at 1 kHz.

The i360 devices were implanted in proximity to the rat L1 anatomical level to interface directly with the spinal cord instead of the existing nerve roots, which are predominantly distal to the L3-L4 level (Fig. 1, E and F). The device was intentionally designed with a narrow profile to ensure it could be placed between two sets of existing nerve roots in the transverse plane, and the device was implanted at the pedicular level of the vertebra, between the superior and inferior exiting thoracic nerve roots. In the axial plane, the device was placed circumferentially around the epidural plane of the spinal cord; for detailed information on the surgical procedure, please refer to Materials and Methods. To detect iatrogenic SCI during implantation of the i360 devices, we performed neuromonitoring at regular intervals during the implantation, which did not show increases in threshold amplitude required to evoke motor evoked potentials (MEP) (*21*). Following the resection of the vertebral laminae, the device was inserted using a leading 7-0 polypropylene suture (Ethicon Inc., Raritan, NJ, USA). After successful device implantation, the electrode located in the midline of the spine was used to calibrate the topographical interpretation of the acquired signals.

### Using the i360 device to stimulate the spinal cord circumferentially

**Fig. 2.**
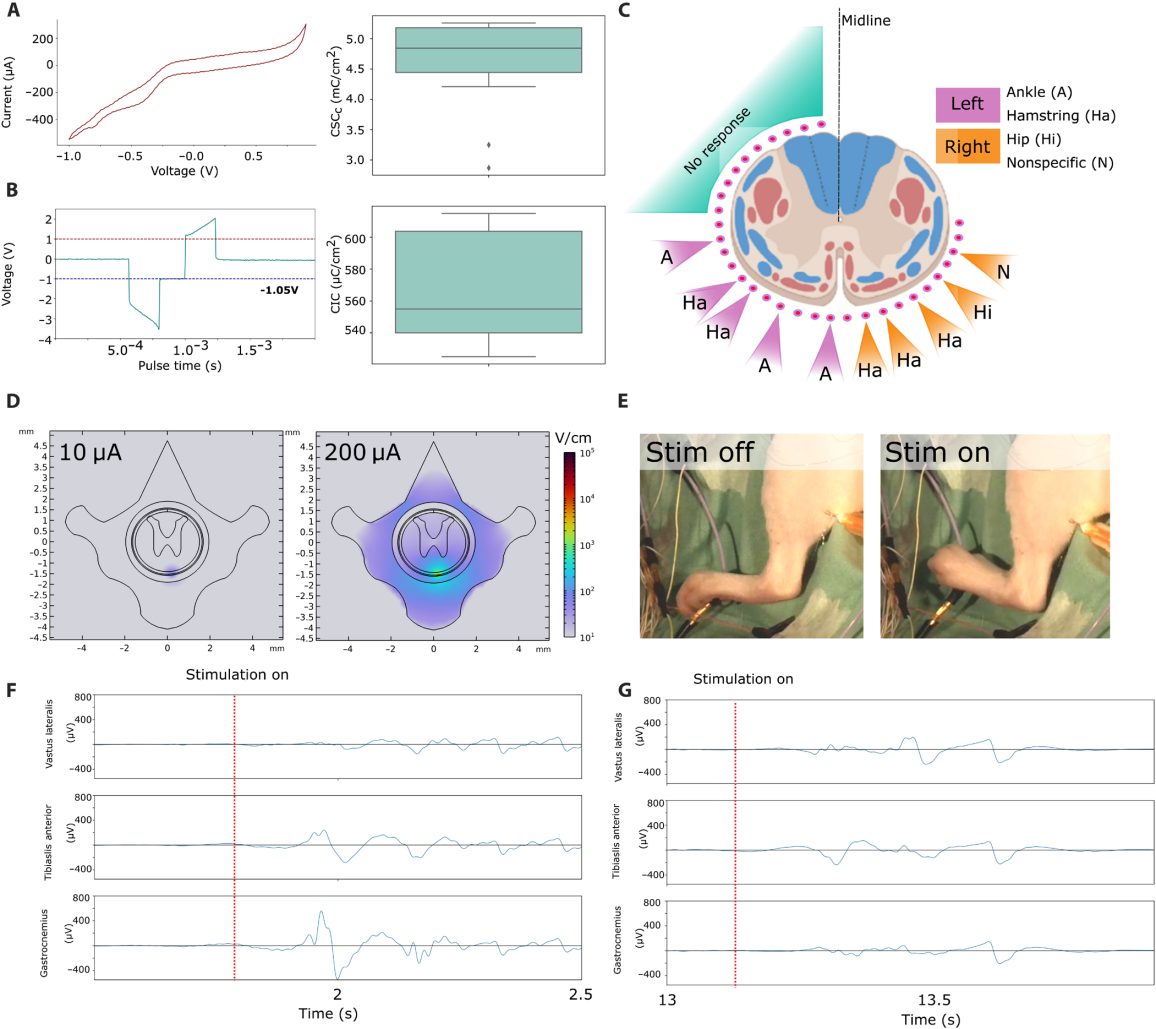
Utilization of the i360 device as a neurostimulator. (**A**) A representative cyclic voltammogram (CV) trace and derived charge storage capacity (CSC) data (*n* = 10). (**B**) A representative transient voltage graph following an electrical pulse and derived charge injection capacity (CIC) data (*n* = 8). (**C**) The outcomes of a stimulation study in a rodent model, illustrating precise control over several lower limb motor groups without affecting the dorsal sections of the spinal cord. In this visualization, red pathways indicate descending tracts, while blue pathways denote ascending tracts. (**D**) Finite element simulation of the electric field magnitude in the rat spine when stimulated with 10 or 200 μA. Note the logarithmic scaling of the color map. (**E**) A segment from a live video recording of lower limb muscle flexion during stimulation of the spinal cord at 20 μA. (**F**) EMG recordings (*n* = 3) of a ventral electrode stimulation. (**G**) EMG recordings (*n* = 3) of a ventral electrode stimulation using an electrode positioned 0.5 mm more laterally. EMG recordings were taken away from the ventral midline, both on the left side. EMG recordings were captured with an Intan RHS unit at 30 ks/s, using needle electrodes in the ankle (Tibialis Anterior), hamstring (Gastrocnemius), and hip (Vastus Lateralis) muscles.

We initially used the i360 for targeted region-specific stimulation of the spinal cord. We investigated the application of SCS around the perimeter of the spinal cord. We were especially interested with the effects of tonic stimulation on the ventro-lateral side of the spinal cord. First, the devices were characterized for charge storage and charge injection capacity. Using microelectrodes, it is important to ensure that the charge delivered is not damaging to the neural tissue. The application of PEDOT:PSS on a conductive metal (Au) electrode markedly increases both charge storage and charge injection capacity (*22*) and allows for wider stimulation limits. These data are summarized in Fig. 2 (A and B), respectively.

We investigated the application of the i360 device to recruit specific motor movements located in the ankle, hamstring, and calf of the animal by localizing stimulation to different portions of the ventrolateral spinal cord circumference (Fig. 2C). The stimulation experiments (Fig. 2, C and E) were supplemented with in silico data (Fig. 2D) looking at a current spread to infer whether stimulation and therefore activation could be localized to specific portions of the cord. The simulations were carried out across multiple peak current levels; here, minimum and maximum currents were used during the in vivo experiments, 10 and 200 μA, respectively; this threshold amplitude was determined as with intraoperative neuromonitoring protocols, which is the lowest amplitude needed to elicit a motor response in the target muscle group simulations reported, and further simulation experiments are shown in figs. S3 and S4. Overall, we observed that stimulation could be localized well for a range of parameters, penetrating into the cord at the site of the active electrode without spreading to nearby regions. These results supported the exploration of motor recruitment selectivity in vivo. Physical properties used to derive these values are outlined in fig. S5. Using tonic stimulation patterns of 50 to 100 Hz with 10 to 20 pulse trains, smooth movements were elicited in a controlled bilateral manner. Ankle, hamstring, calf, hip, and flank muscles were recruited, as shown in Fig. 2E. Electromyography (EMG) data were collected from the gastrocnemius (calf), tibialis anterior, and vastus lateralis (quadricep) muscles (Fig. 2, F and G) using steel needle electrodes. For each electrode, three recordings were taken and overlaid, showing muscle recruitment differences between two ventral electrodes separated by 0.5 mm. Within each experiment, we confirmed that stimulation of the dorsal spinal cord circumference at the amplitude parameters used in the ventrolateral portion produced no movements.

### Using the i360 device to record spinal signals circumferentially

**Fig. 3.**
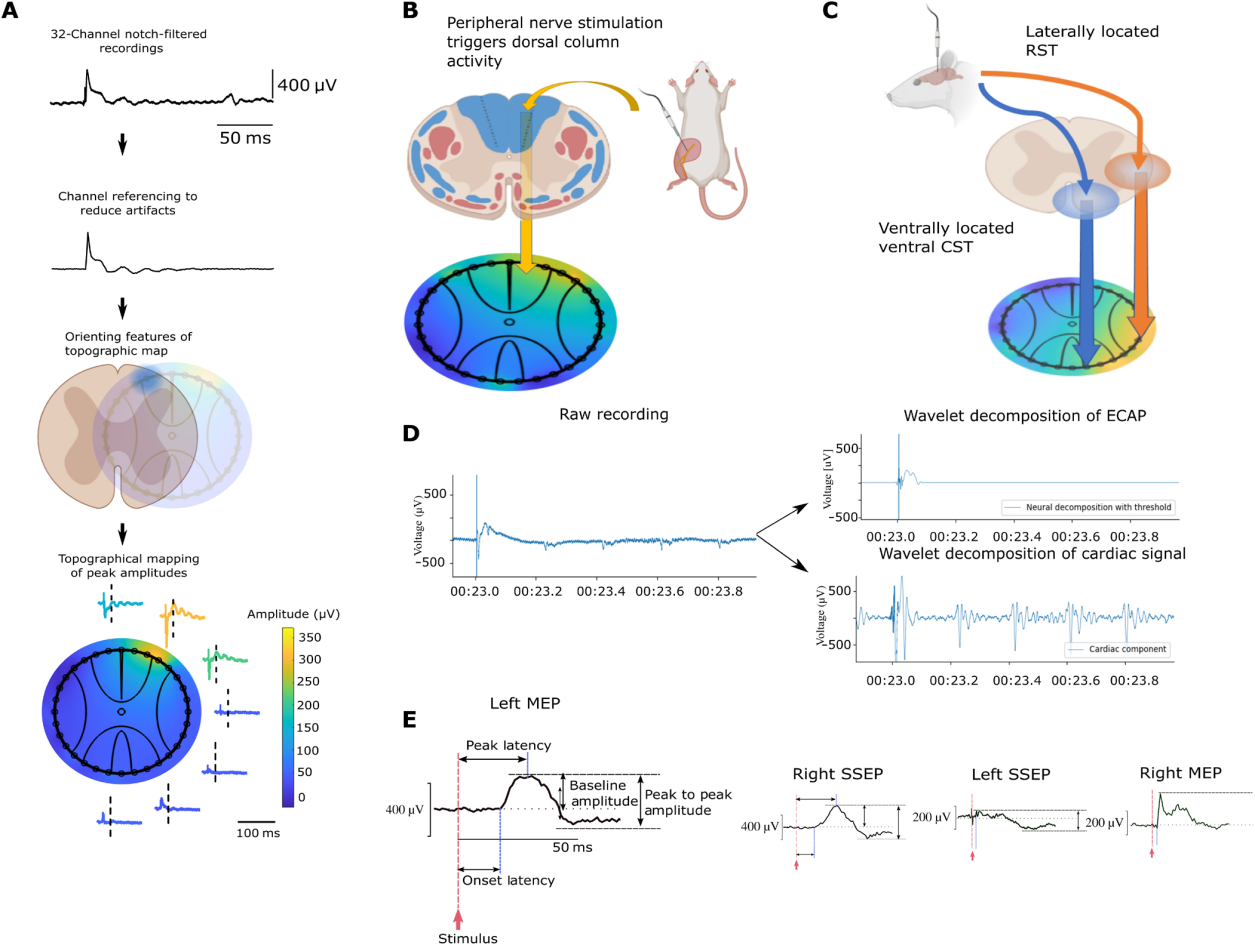
Circumferential spinal cord recording enhances topographic representation. (**A**) Process flow of topological map creation: First, recording of raw signals from 32 channels around the spinal cord. Second, channels are referenced and filtered to remove artefacts. Third, features are orientated on a map based on electrode position and coverage. Fourth, a topological representation of circumferentially recorded epidural signals was created based on the peak amplitude of neural signals. (**B**) Topological map created from SSEP recordings demonstrated a peak amplitude hotspot on the dorsal area of the spinal cord. (**C**) MEP recordings demonstrated peak amplitude hotspots predominantly on the ventrolateral and lateral areas of the spinal cord corresponding to approximate locations of the ventral corticospinal tract (CST) and rubrospinal tract (RST), respectively. (**D**) Wavelet decomposition of an evoked signal leads to extraction of the cardiac and neural components of the raw signal. (**E**) Recorded compound action potentials can be analyzed in greater detail; here, details of waveform analysis are provided. Both left and right SSEP and MEP waveforms from representative electrodes on the spinal cord are shown.

We then used the i360 device to record neural signals around the spinal cord. Following safe implantation of the devices as evidenced by preserved hindlimb MEP responses, we performed acute electrophysiology by triggering the sequential activation of bilateral MEP via the hindlimb motor cortex and somatosensory evoked potentials (SSEP) via the sciatic nerve, which were recorded by the i360 device. The circumferential placement of the electrodes around the spinal cord enables a comprehensive topographic representation of the acquired signal amplitudes at each sampled interval (Fig. 3A). This spinal cord topography capitalizes on the ordered arrangement of ascending and descending tracts traversing the spinal cord. This is illustrated when stimulation of the left sciatic nerve produces a hotspot on the spinal cord topographic map corresponding to the expected location of the dorsal column (Fig. 3B).

The density of electrode recordings around the spinal cord also allows better differentiation of various hotspots around the spinal cord. The descending motor pathway is mediated by the rubrospinal and corticospinal tract in rats (*1*, *23*, *24*). This is substantiated by the spinal cord topography maps generated in Fig. 3C, where two locations of peak activation are highlighted simultaneously following MEP stimulation; this would correspond to the approximate location of these descending motor axons.

Besides using channel referencing to isolate neural signals, we were able to automatically isolate neural signals from instrumental and biological noise generated by electrocardiogram (ECG) signals and respiratory activity using wavelet decomposition as a denoising technique (Fig. 3D) (*25*). Using either method of neural signal extraction allows the study of signal waveforms to obtain conventional measurements of latency, amplitude, and neural conduction velocity in a low-noise environment (Fig. 3, E and F).

### Analysis of circumferential spinal cord recordings

**Fig. 4.**
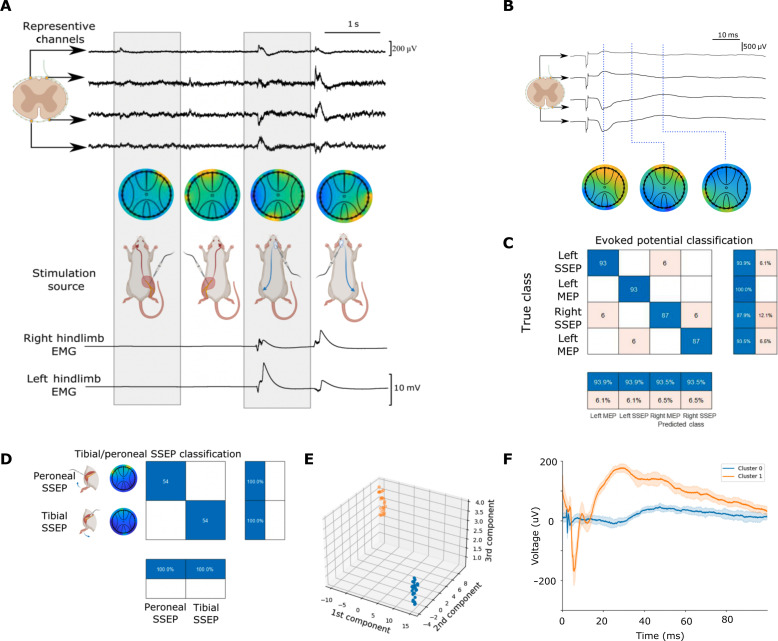
Analysis of recorded spinal signals. (**A**) Representative recordings across four quadrants of the spinal interface covering left dorsal, left ventral, right dorsal, and right ventral areas of the spinal cord during SSEP and MEP stimulation events. Topographic heatmaps at a single point in time, as well as associated EMG recordings, are provided. (**B**) A diagram from the four representative channels demonstrating how recorded signals evolve through time following stimulation of the sciatic nerve; these can also be represented temporally as in fig. S6. (**C**) A matrix of classification showing positive and negative hit rates of left/right SSEP/MEP stimulation events. (**D**) A classification matrix showing tibial versus peroneal stimulation events. (**E**) A three-dimensional graphical representation of the principal components analysis (PCA) showing how tibial and peroneal stimulation can be clustered based on waveform shape for a single dorsally positioned electrode, an expanded version of this is shown in fig. S8. (**F**) Temporal evolution of the averaged clustered waveforms (trace) and 1 SD (trace shadow) for a tibial versus peroneal stimulation event from a single dorsally positioned electrode, an expanded version of this data is shown in fig. S9.

Integrating the abundant neural signal data in both the time and spatial domains allows for the identification of unique topographic signatures that reflect the dominant spinal cord tract activated. We produced sequences of alternating left and right MEP and SSEP stimulation, producing the patterns corresponding to the expected location of spinal cord tract activation. This allows clear visualization of where the tracts are activated and produces unique topographic signatures that allow for accurate classification of the source signals (Fig. 4A).

The high sampling rate of recordings obtained also enables the interrogation of peri-event potential amplitudes following an evoked potential, highlighting areas of shifting peak amplitudes around the spinal cord. In our evaluation of the topographical data following SSEP stimulation of the sciatic nerve, we were able to identify multiple signal peaks consistent with previous studies (*26*, *27*). These peaks, however, were of different relative amplitudes in the various channels, allowing us to demonstrate that the topographic hotspot shifts with time after stimulation (Fig. 4B). This phenomenon was reflected in most of our recordings, and we postulate that this shift reflects complex interneuron interactions with dorsal column neurons activated and subsequently sending impulses to ventrally based corticospinal and spinothalamic neurons.

The circumferential spinal cord recordings generate topographic peak signal amplitudes distributed over the 32-channel recordings at each sampling interval unique to the various sources of evoked potentials. Using a thresholding method based on the peak amplitude means allows feature extraction of the signals in preparation for data analysis. This allows for the use of a supervised machine learning approach to identify these characteristics to classify the source of evoked potentials using a *k*-nearest neighbors (KNN) algorithm. This resulted in a classification accuracy of 93.8% when categorizing source signals according to the following categories: left MEP, right MEP, left SSEP, and right MEP (Fig. 4C).

The density of signal information obtained from the circumferentially placed electrode also enabled further differentiation of the source signal to the level of peripheral nerve branches. The tibial nerve, supplying and receiving sensorimotor input from the anterior compartment of the leg, was stimulated in an alternating sequence, with the peroneal nerve reflecting the posterolateral compartment of the leg. The stimulation of these two sciatic nerve branches generated unique spatial patterns represented by the 32 channels on the electrode, allowing the classification of the source signals (Fig. 4D).

An automated strategy to extract the signal features for classification was also investigated, using linear and nonlinear dimensionality reduction methods. Following wavelet decomposition to identify cardiac and neural peaks (fig. S7), this allowed an unsupervised machine learning classification algorithm to identify distinct clusters allowing comparable classification accuracy of tibial/peroneal nerve stimulation source signals, albeit at a higher computational cost and processing time. This allows for a secondary method of classifying peroneal and tibial alternating stimulation, as shown in Fig. 4E; a representative waveform comparison from a dorsally located electrode is shown in Fig. 4F.

### Bypassing complete SCI: Proof of concept

**Fig. 5.**
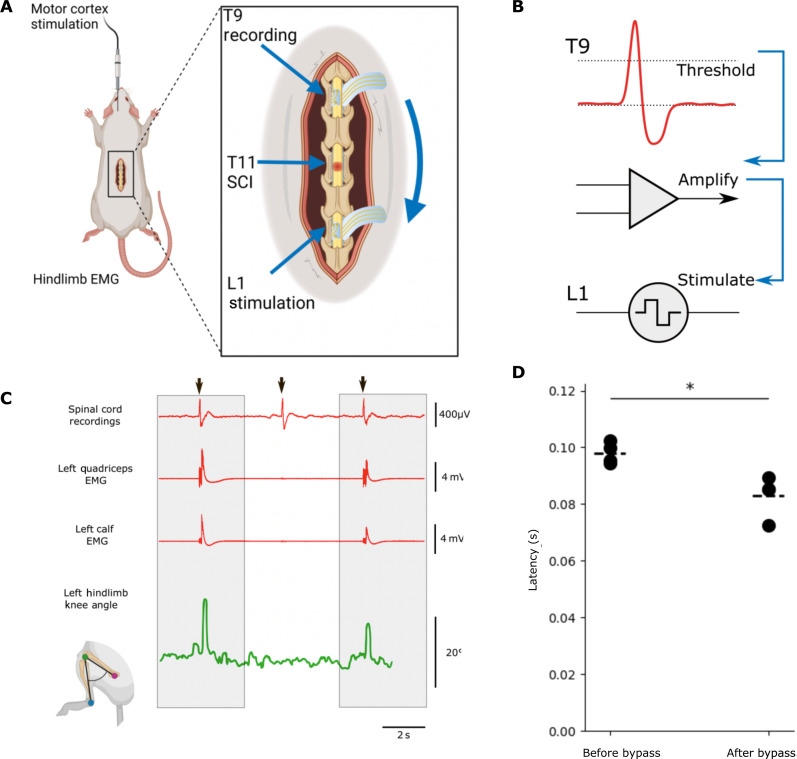
Circumferential spinal cord interfacing could enable a bioelectronic bypass in acute SCI. (**A**) In the experimental setup for the spinal bypass experiments, one device is positioned above the injury, and one device is positioned below the injury. Intramuscular EMG probes are connected to the lower limbs. MEPs are generated using a microwire placed in the motor cortex. (**B**) A diagram of the recording–stimulation workflow, a signal is recorded at T9, amplified, and an amplitude threshold is applied and used to stimulate a corresponding electrode below the injury. (**C**) Example data showing recorded signals from the ventral part of the spinal cord and quadricep/calf EMG waveforms following sub-injury stimulation. Hindlimb angles were calculated from markerless kinematic data, which correspond to EMG and MEP waveforms. (**D**) MEP latency calculated before (0.09797 ms; SD, 0.01256 ms) and after (0.08308; SD, 0.02009 ms) the SCI was established (*n* = 4). Two-tailed *t* test, *P* value < 0.05 (0.0112).

We exploited both the recording and stimulating capabilities of i360 by using neural signals recorded around the spinal cord as triggers to produce targeted SCS to functionally bypass the site of acute SCI. This approach required the implantation of two i360 devices at the T9-T10 and T12-L1 levels, respectively, using neuromonitoring to ensure that the implantation did not lead to an iatrogenic SCI. An acute transection of the spinal cord was then performed between the two devices, with the lack of MEP recorded in peripheral EMG traces demonstrating the discontinuation of descending motor signals (Fig. 5A).

To produce a low-latency communication between the recording and stimulating device, we used an evoked compound action potential threshold detection method that was not computationally intensive. This allows the proximal, recording i360 device to detect the peak of the MEP action potential and trigger the distal, stimulating the i360 device to affect hindlimb movement (Fig. 5B). The electronic bypass can be deactivated and reactivated readily to allow differentiation between states of rest and activity. For this technique to function as a physiological bypass, the latency between the recording and stimulation algorithm should aim to be comparable to the latency of neural transmission along the spinal cord (Fig. 5, C and D). Before the installation of the bioelectronic bypass, the mean spinal cord conduction latency was 0.098 ± 0.013 ms. Upon implementing the bypass, we observed a decline in mean latency to 0.083 ± 0.020 ms. A two-tailed *t* test generated a *P* value of 0.0112. This confirms a statistically significant reduction in latency following the bypass implementation, as illustrated in Fig. 5 (C to E). The bypass system outperformed our initial objective of equalling, providing a promising outlook for the viability and potential of our technique.

**Fig. 6.**
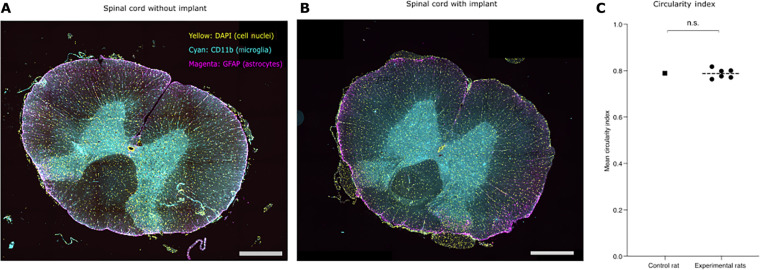
Chronic device implantation—Preservation of spinal cord circularity in freely moving animals. (**A**) Immunofluorescence imaging of the spinal cord from a control rat. (**B**) Immunofluorescence imaging of the spinal cord from a rat implanted with a dummy device. The device was not linked to a headstage/head cap, and no stimulation was administered. (**C**) Scatterplot comparing the mean circularity index of experimental and control rats [one-sample *t* test, *P* value = 0.8661, 95% confidence interval (0.7658, 0.8092)]. Scale bars, 100 μm. DAPI, 4′,6-diamidino-2-phenylindole; n.s., not significant.

**Fig. 7.**
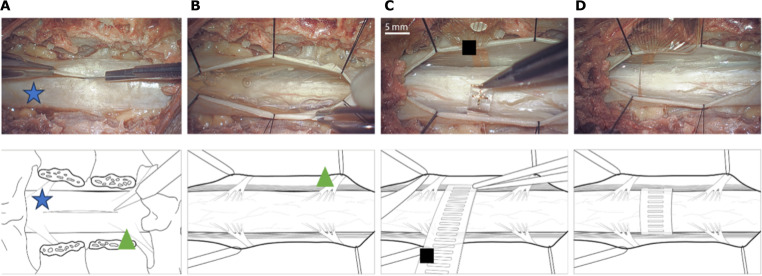
Early human translation of the i360 device using a human cadaver (pictures and illustration). (**A**) A standard midline durotomy following spinal cord reconstitution and laminectomy. (**B**) Gently retracting the dura using sutures to improve visualization of the lateral aspect of the spinal cord and exiting roots. (**C**) Feeding the human-scaled i360 dummy device through the lateral aspect of the spinal cord. (**D**) The device is shown encircling the spinal cord. Blue star, dura; green triangle, spinal nerve root; black square, I360 device.

To evaluate the potential for iatrogenic injury resulting from chronic implantation of the i360 device in rats, we conducted a study involving the implantation of a dummy (not electrically active) i360 device in six rats. The device was left in place for up to 6 weeks. Throughout this period, we monitored the animals’ well-being and motor function. Experimental animals were terminated at six different endpoints. The spinal cord was carefully dissected and examined for signs of trauma or deformation. Microscopic examination revealed that the spinal cord has preserved its inherent morphology and its staining features (Fig. 6, A and B), while the animal’s mobility remained unimpaired. Spinal cord circularity index—a metric to quantify mechanical deformation and damage of the spinal cord in spinal cord device implantation (*28*, *29*)—was calculated for each rat and summarized in Fig. 7C. No significant difference in the mean circularity index between experimental and control rats was identified (Fig. 6C).

### A step toward human translation and chronic implantation

Last, we scaled the i360 to human dimension to explore surgical implantation feasibility. A parylene-C–based dummy device was used, that is, a device with the same dimension and mechanical characteristics as a human-scaled i360 but with none of the electrical connections required to function as an electrical interface. First, the human spinal cord was reconstituted using a saline pump, this involved inserting and sealing a fluidic tube intradurally and connecting it to a flow pump, maintaining a controlled pressure environment for 30 min preimplantation, as previously reported (*14*). A laminectomy was performed at the thoracic T9 to T10 level by two experienced spinal surgeons. Following this, a midline durotomy was performed to expose the parenchyma, as illustrated in Fig. 7A. The dura was gently retracted using tenting sutures to allow the surgeon to operate in the space (Fig 7B). Figure 7C shows the i360 being passed laterally down the side of the spinal cord. Last, Fig. 7D shows that the device is wrapped around the spinal cord, completing the surgery. Such a strategy, which involves maneuvering synthetic dural material around the spinal cord, is routinely used for the repair of ventral dural defects, which often result from thoracic disc herniations (*30*, *31*). When using an electrically active device at this point, the interface could be used to record signals from or stimulate the spinal cord.

## DISCUSSION

In this study, we have demonstrated that interfacing circumferentially around the spinal cord enables safe recording and stimulation and allows a comprehensive representation of neural signals. While recent efforts have focused on the use of SCS in rehabilitation following incomplete SCI, the approach described here has the potential to electronically bypass a complete SCI. The ability to trigger targeted and direct SCS and record from each spinal cord tract will facilitate the creation of a bypass when combined with physiologically relevant latency.

This technique was made possible by harnessing the advances in thin, flexible, and organic bioelectronics that can be implanted around the spinal cord without causing neural damage. The techniques are made possible using nonpenetrating arrays, which limit injury when compared to stiff and bulky current SCSs and minimize foreign body reaction secondary to chronic implantation (*32*).

Previous methods of circumferential spinal cord recording used the use of penetrating electrodes (*11*, *12*), with advances in electrode fabrication allowing the implantation of multiple electrode arrays to increase the area of simultaneous neural recording (*33*–*35*). Penetrating electrodes, however, can lead to SCI along the electrode insertion tract and, translating to chronic applications, faces the issue of wire breakage (*33*) and elastic modulus mismatches leading to chronic neural injury (*36*). Epidural electrodes minimize iatrogenic SCI (*37*) while recording information in the ascending and descending tracts of the spinal cord, located peripherally in the white matter. Previously, epidural recording of the ventral and ventrolateral tracts was challenging due to the anatomical obstacles (vertebral body and neural arch) preventing the safe insertion of recording probes without causing SCI, restricting the investigation of spinothalamic and ventral corticospinal tracts, the latter of which is the dominant motor pathway in humans. The surgical technique described in this work, adapted from established techniques used in routine clinical practice, overcomes these anatomical obstacles by judicious resection of the neural arch to slide the i360 devices safely under the ventral spinal cord. Although a necessary part of the implantation process for a rodent scale device, this is not required for human placement due to the greater size.

Owing to the somatotopic arrangement of hindlimb muscle groups around the spinal cord, we were able to achieve selective activation of specific hindlimb muscle groups. This was achieved by directly stimulating the ventral and lateral epidural spinal cord, where the ventral corticospinal tract and anterior motoneurons are located. In addition, the pattern, intensity, and duration of muscle activation can be modulated by varying the stimulation parameters to produce gentle, sustained, or explosive movements (*38*). This can potentially empower neuroprosthetics to enable movements beyond the current walking or stepping-targeted SCS algorithms, allowing patients with SCI to perform specialized activities. This is especially exciting given the relatively modest number of electrodes (32 contacts) used here, as increasing this number will improve classification and fidelity. Moreover, acknowledging that a more complex computational algorithm may increase the bridge’s latency, it is important to note that the enhanced signal capture offered by our technology may reduce the necessity for elaborate computation, thus potentially mitigating the increased latency by simplifying the decoding of motor activity and volition.

While conducting active chronic experiments in rat models is technically feasible, we believe that it is more pragmatic and scientifically relevant to transition to larger animal models mainly due to the anatomical and physiological similarities of these to human (*20*). We previously reported on high-density thin-film electrodes (*39*), and our future efforts will focus on exploring chronic applications in large animal models.

Accompanied by advances in biomaterials and signal processing, neuroprosthetics can potentially restore volitional motor function to patients with SCI and act as a key tool during rehabilitation or prognostics, especially for those suffering from an incomplete injury. We have demonstrated that trigger signals for targeted SCS can be generated by direct spinal cord recordings using a low-latency electronic bypass, controlling hindlimb motion in acute SCI. This was made possible by the circumferential implantation of a thin, flexible device with high electrode density to allow comprehensive representation and stimulation of the spinal cord. Further work is required to validate this bioelectronic bypass strategy in chronic and large animal models before clinical trials. One concern is the stability of thin-film electrodes, which is now being addressed by tuning materials and device structure. Accelerated aging studies show that by replacing the Au tracks with PEDOT:PSS, electrode stability is increased from ~9 months to ~6 years (*40*). Further improvements can be achieved by, e.g., replacing parylene with silicone (*41*). We envision that direct interfacing with the spinal cord represents a viable strategy for bypassing a site of injury in the spinal cord, restoring volitional motor function to patients with SCI.

## MATERIALS AND METHODS

### Device design and fabrication

A 32-channel staggered linear array electrode was designed to record epidural spinal cord neural signals. This design is intended to maximize the circumferential representation of the signals while minimizing cross-talk from electrode proximity with its staggered configuration. The interconnects are minimized to reduce its footprint, allowing for a narrower device that can be implanted without excessive dissection around the spinal cord. The device is appended by a reinforced gold ring that allows a size 7-0 polypropylene suture (Ethicon Inc., Raritan, NJ, USA) to pass the device through the ventral aspect of the spinal cord. The device was prepared in several sizes (8.90, 9.90, and 10.90 mm in lengths) to anticipate variations in rat spinal cord anatomy and to distribute the electrodes optimally around the spinal cord (*19*, *20*). The electrode size was 0.1 mm by 0.1 mm in a square configuration.

The device was fabricated using standard photolithography techniques (*16*). The first parylene deposition is performed using the PDS 2010 Labcoter 2 (Specialty Coating Systems Ltd., Woking, UK) on a 10.16-cm silicon wafer (Microchemicals GmbH, Ulm, German), with 4 g of parylene-C dimer to form layers of 2 μm. This is followed by the first photolithography for the gold electrode patterning using the MA/BA6 mask aligner (SÜSS MicroTec, Garching, Germany), spin coating the AZ nLOF 2035 negative photoresist (MicroChemicals GmbH, Ulm, Germany) at 500 and 1000 rpm for 5 s and at 6000 and 8000 rpm for 30 s to achieve a thickness of 2.00 to 2.50 μm, followed by soft baking at 110°C for 60 s, ultraviolet light exposure for 7 s, and postbaking at 108°C for 150 s. AZ 726 MIF (MicroChemicals GmbH, Ulm, Germany) is used as a developer for 20 to 30 s before distilled water washing and drying with compressed nitrogen.

The samples are activated using oxygen plasma under vacuum (60 s at 100 W and 0.6 mbar O_2_) using the Plasma Pro 80 RIE (Oxford Instruments Plasma Technology, Bristol, UK) to improve adhesion between parylene and the electronic components, titanium (Ti) and gold (Au) that were deposited via electron beam evaporation (Kurt J. Lesker Company, Jefferson Hills, PA, USA). Ten-nanometer Ti was evaporated under vacuum (<6 by 10^−6^ torr) and allowed to rest for 10 min, followed by 100-nm Au deposition. The sample is then left in acetone for 30 to 60 min and subsequently lifted off carefully with a swab stick. Last, they are rinsed with isopropyl alcohol and deionized water.

The second layer of parylene is deposited using the same steps described above. This is followed by photolithography for the outlines of the devices using the MA/BA6 mask aligner (SÜSS MicroTec, Garching, Germany), spin coating the AZ 10XT (MicroChemicals GmbH, Ulm, Germany) negative photoresist at 500 and 1000 rpm for 5 s and at 3000 rpm for 30 s to achieve a thickness of ~6.00 μm, followed by soft baking at 110°C for 120 s, ultraviolet light exposure at six steps of 5 s with a 5-s interval between the steps. The sample is subsequently developed in AZ 726 MIF (MicroChemicals GmbH, Ulm, Germany) developer for 6 to 7 min and washed with DI. Reactive ion etching (RIE) is then completed with the Plasma Pro 80 RIE (Oxford Instruments Plasma Technology, Bristol, UK) at an etching rate of 200 nm/min. This is followed by the third layer of 2-μm sacrificial parylene deposition separated by soap (2% Micro-90 Cleaning Solution, Cole-Parmer Instrument Company Ltd., St. Neots, UK), followed by further photolithography to expose the electrodes and contact pads using AZ 10XT (MicroChemicals GmbH, Ulm, Germany), and spin-coated for 30 s at 3000 rpm followed by baking for 120 s at 110°C. The samples were then exposed and developed, followed by RIE as described above, until metal exposure was completed.

The conducting polymer PEDOT is combined with PSS, which acts as a counterion to PEDOT to increase the conductivity of the electrodes (*17*). The PEDOT:PSS is prepared according to previously described methods (*42*) with 19 ml of CLEVIOS PH 1000 PEDOT:PSS (Heraeus Holding, GmbH, Hanau, Germany) mixed with 1 ml of ethylene glycol which improves film conductivity and two drops of 4-dodecylbenzenesulfonic acid to increase the homogeneity of the spin-coated film. The mixture is ultrasonicated for 10 to 15 min and filtered through 1.2-μm polytetrafluoroethylene filters. This is followed by spin coating of the PEDOT:PSS layer at 3000 rpm for 30 s, baking for 60 s at 110°C, and 2 cycles of spin coating at 1500 rpm for 30 s and baking at 110°C for 60 s. Last, the sacrificial parylene layer is peeled off to reveal only the PEDOT:PSS layer, and the samples are hard baked for 1 hour at 130°C. The devices were packaged using 33-channel flat flexible cables (Mouser, UK) with a bonder (Fineplacer, Finetech GmbH & Co. KG, Berlin, Germany) using a 5-μm anisotropic conductive film (ACF, Jetro, Japan).

### Electrical characterization

All impedance measurements were taken using an AutoLAB potentiostat, a phosphate-buffered saline (PBS) solution (0.01 M, Sigma-Aldrich), and a platinum electrode was used for both counter and reference. Charge storage capacity of the device was determined using three-point cyclic voltammogram (CV) techniques. Measurements were carried out using an AutoLAB potentiostat with a PBS solution (0.01 M, Sigma-Aldrich) used as an electrolyte. A platinum electrode was used as the counter electrode, while an Ag/AgCl electrode was used as a reference. CV measurements were carried out using a 0.03-V sweep rate and a 0.00244-V step within a window of −1.05 to 0.9 V. A minimum of 6 cycles were performed for each measurement to allow the recording to settle, and only the final recorded cycle was analyzed.

The charge injection capacity of the device was determined using a stimulation source and measured voltage transients using an oscilloscope. An Intan RHS 128-channel stim/recording controller and an RHS2000 32-channel headstage were used to connect to the device and provide a charge-balanced current stimulation pulse with a phase width of 300 μs and an interphase delay of 100 μs. Platinum probes were used as both counter and reference electrodes, and a PBS solution (0.01 M, Sigma-Aldrich) was used as an electrolyte. The pulse was gradually increased in amplitude while observing the voltage transient trace from a Keysight DSOX1204G oscilloscope. When the cathodic interphase transient reached −1.05 V, the current amplitude was recorded. Charge per area was calculated from this current and pulse duration. Multiple devices were characterized (*n* = 3) from separate device batches for each test.

### Animal preparation

Sixteen adult female Sprague-Dawley rats (Charles River Laboratories, UK) weighing 200 to 250 g were used for acute electrophysiology experiments. The animals were anesthetized with intraperitoneal urethane (1.3 g/kg) with subcutaneous buprenorphine (0.05 mg/kg) analgesia, and liquid gel eye lubricant was applied. Urethane was selected as the anesthetic agent as it minimally depresses evoked sensorimotor responses when compared to volatile anesthetic agents (*43*). The temperature of the animal was monitored constantly and kept between 37° and 38°C via a self-regulating heating pad. The animal was secured to a stereotaxic frame, keeping the cervical spine neutral with an elevated operating frame. The frame is customized to keep the abdomen free to decrease venous congestion and subsequent bleeding during surgery. All animal husbandry and procedures were performed in accordance with the UK Animals-Scientific Procedures Act (ASPA) 1986 and under the approval of the animal welfare and ethical review body (University of Cambridge).

Craniotomy was performed over the expected hindlimb motor regions with established techniques described in previous studies field (*36*) to allow MEP generation via intraneural microwires. Care was taken to prevent the disruption of the subdural vessels and underlying parenchyma. The sciatic, peroneal, and tibial nerves were exposed via previously published techniques (*44*–*46*). Animals used for acute electrophysiological experiments were euthanized at the end of the session using pentobarbitone (200 mg/kg intraperitoneal) and cervical dislocation.

### Surgical technique (rat in vivo studies)

The thoracolumbar spine was exposed via a 4-cm incision from T11-L1, using the last palpable rib as a surface landmark. After laminectomy (T12-L1 level) exposing the dorsal epidural surface of the thoracic spinal cord, the pedicles of the vertebrae are resected with a rongeur to the ventral base of the pedicle, providing access to the ventral epidural plane. The ventral epidural plane is then developed with a blunt dissector to the contralateral pedicular base, allowing the passage of the blunt end of a 7-0 polypropylene suture. This preparatory step minimizes the inadvertent laceration of the ventral dura. The passed suture is then secured to the leading end of the electrode array, and gentle traction is applied to guide the array across the ventral epidural plane. The level was chosen as it is proximal to the end of the spinal cord at the rat L3-L4 region field (*47*), and recording at this level will be able to capture the sensorimotor signals presented to the hindlimb nerves without excessive noise from the intercostal muscles or from echocardiogram recordings.

Neuromonitoring was performed with MEP recordings at regular intervals and after key procedural steps to detect any deterioration in signals, ensuring that a barely observable hindlimb movement was present at the same threshold stimuli. The neural arch must be resected to the base of the vertebral body to visualize the lateral aspects of the spinal cord and the existing roots. The spinal cord is then evaluated to determine the ideal device size to maximize signal recording coverage.

The selected device is then secured with a double-ended 7-0 polypropylene suture (Ethicon Inc., Raritan, NJ, USA) to pull the device under the spinal cord. To prevent iatrogenic dural injury, the needle was inserted via the blunt end in the ventral epidural plane, taking care to guide the tip against bone to prevent encroaching on the spinal cord. Once passed, the blunt end is retrieved, and the procedure is repeated with the other end of the suture to gently pull the device across the ventral spinal cord while preventing the twisting of the device. Once across, the end of the device is tucked under itself over the dorsal spinal cord, ensuring circumferential coverage of the spinal cord. The electrode located in the midline of the spine is used to reference the rotational orientation of the device, and the position of the device is secured with silicon sealant (Kwik-Sil, World Precision Instruments, Hitchin, UK).

For chronic implantation of devices into the spinal cord, animals were recovered following implantation and monitored for motor dysfunction. We used The Basso, Beattie and Bresnahan scale for locomotor scoring. One animal developed motor disability 3 days after implantation associated with spinal instability. For subsequent animals, the spinous processes of vertebrae above and below the site of implantation were fused with fine steel wire and surgical cement. No further motor dysfunction associated with spinal instability was observed. Animals were implanted and monitored individually at progressively longer time points, up to a maximum of 6 weeks, due to regulatory limitations to minimize the likelihood of animals developing motor dysfunction or other unforeseen surgical complications.

For acute spinal cord electronic bypass experiments, an additional recording I360 device was inserted at the T9-T10 level using the same technique. After MEP stimulation to confirm the continuity of the descending motor circuitry, an acute SCI injury was created at the intervening T11 level using a sharp no.15 blade (Swann-Morton, Sheffield, England) going through the spinal cord completely. A 2 mm x 2 mm x 2 mm piece of cellulose sponge (Fine Science Tools GmbH, Heidelberg, Germany) is then inserted at the gap to arrest bleeding and ensure discontinuity of the spinal cord.

### Stimulation modeling

The electric field distribution in the rat spine was analyzed by conducting a three-dimensional finite element simulation using COMSOL Multiphysics 6.0 (electric current interface of AC/DC module), solving the point form of Ohm’s law, assuming current conservation. We simulated fixed DC electrode injection currents of 10 or 200 μA at the electrode surface. Our model geometry is a three-dimensional extension of the two-dimensional geometry, which is based on magnetic resonance imaging scans of rat T10 vertebrae (*48*). The geometry consists of muscle, bone, epidural space, dura, cerebrospinal fluid, and white and gray matter domains. The device is represented by a 4-μm-thick ring of a low conductivity (0.001 S/m) material representing parylene-C with (100 μm by 100 μm)–sized electrodes placed directly on the dura surface. All domains were approximated to be perfectly isotropic conductors. The material conductivities can be found in the Supplementary Materials. The spine model was embedded in a (50 by 50) mm^2^ cube with the electrical conductivity of muscle tissue and with grounded outer boundaries. The Supplementary Materials contains the results of a stability analysis showing that the simulation results are independent of the box size and spine length. The figures in this article show the electric field magnitude in the plane normal to the electrodes.

### Acute electrophysiology

The recording I360 device was connected to a 32-channel recording headstage (Intan Technologies, Los Angeles, CA, USA) via a custom-built omnetics/ZIF connector printed circuit board (PCB) and flexible flat cable; signals were acquired at 30 kHz through the RHS 2000 stimulation/recording controller (Intan Technologies, Los Angeles, CA, USA) with a 50-Hz notch filter to reduce line noise from electric current sources. The device was grounded via a stainless-steel wire implanted into the paravertebral muscles, and impedance measurements were obtained.

MEP stimulation was performed using a 50-μm tungsten microwire electrode insulated with polyamide (California Fine Wire Company, CA, USA) secured to a stereotaxic frame and grounded to a stainless-steel cerebellar screw. The microwire was guided to the cortical areas representing hindlimb motor function as determined by previous mapping studies (*49*). Motor cortex stimulation was performed with a 200-Hz train of 20 alternating square wave pulses of 100-ms pulse width to maintain a short stimulation duration (*50*), starting from 50 μA and increasing in 25- to 50-μA steps until the minimum stimulus energy needed to elicit a barely perceptible muscle contraction, referred to as the threshold level, was determined (fig. S1). If a satisfactory MEP was not obtained, the area of motor cortex stimulation was shifted 3 to 5 mm in depth and/or rostrocaudal direction.

SSEP stimulation was performed using a customized nerve hook electrode applied over the sciatic, peroneal, and tibial nerve, anchored via silicon sealant (Kwik-Sil, World Precision Instruments, Hitchin, UK) and grounded to a stainless-steel wire placed at the base of the tail. The stimulation protocol consisted of a single alternating square wave pulse of 100 ms, starting at 50 μA and increasing in 10- to 25-μA steps until the threshold level was determined (fig. S1).

EMG was recorded using electrodes made of stainless steel 25-gauge needles, inserted into the quadriceps muscles and grounded to a stainless-steel wire placed at the base of the tail. Trains of alternating left/right, SSEP/MEP stimulation were applied to provide data for analysis and machine learning, lasting approximately 70 s per session with 1 s between each stimulation pulse. All stimulation protocols were applied using the RHS 2000 stimulation/recording controller (Intan Technologies, CA, USA).

### Signal processing and topographical plot

The raw signals were imported into MATLAB (R2021a) without further filtering to preserve key time domain information. Active channels were referenced to dormant counterparts to reduce ECG, respiration, stimulation, and movement artifacts. ECAPs were identified, defined as time-locked positive waveforms following a stimulus (*51*), using an automated peak finder and verified by visual inspection of the waveforms to ensure that erroneous signals were not included in the analysis. As the signals in the 32 channels were of varying amplitudes at each sampled interval, the amplitudes in each channel around the spinal cord could be plotted in a topographic heatmap using a custom MATLAB script (online repository: 10.5281/zenodo.10908887), visualizing the areas of activation at each sampling interval. This topographic heatmap generated a spatial signature that approximately corresponded to the area of underlying spinal cord tract activation and could be used to correlate with the source of the evoked potential.

### Wavelet decomposition and spike detection

A subset of the raw recordings was independently analyzed using wavelet decomposition to validate the ability of this method to automatically isolate the neural signals from biological and instrumental noise, reducing the manual workload that accompanies the previously described processing. A direct approach to identify the ECAPs was taken by performing a denoising multiresolution analysis of the signal using discrete wavelet transformation as originally proposed by Diedrich *et al.* (*25*) as this choice outperforms other alternatives when working with similar neural signals. Orthogonal Daubechies of order 5 using three levels of decomposition were empirically chosen on the basis of their similarity to the ECAPs of interest and to filter out low frequencies. The hard-thresholding regular wavelet denoising technique proposed by Donoho and Johnstone (*52*) was chosen, which essentially applies a threshold to the coefficients at each decomposition level in the orthogonal time-frequency domain to further isolate the structures of interest before transforming them back into the original domain denoised. The threshold was calculated as follows

$$\text{Th}=\sigma \;\sqrt{2\;\text{ln}\left(N\right)}$$ (1)

where σ is the SD of the Gaussian noise, and *N* is the number of samples in the coefficients. Because orthogonal wavelets were used, the length of the coefficients at all decomposition levels is the same as in the original signal. At each level “*l*,” the σ was calculated as

$$\sigma_{l}=\frac{\text{median}\left(|c_{l}|\right)}{0.6745}$$ (2)

where *c_l_* was the value of the wavelet detail coefficients at each level, and 0.6745 is the 75th percentile of the standard normal distribution (*52*). Then, detailed coefficients were hard-thresholded for the reconstruction of the denoised signal as

$$y=\left\{ \begin{array}{c} x\;\text{if}\;|x|>\text{Th}\\ 0\;\text{if}\;|x|<\text{Th} \end{array}\right.$$ (3)

Spikes on the reconstructed signals were then identified by using a detection threshold as defined in Eq. 1. A spike window centered around the detected peaks of 4-ms length was extracted to obtain the corresponding waveforms. The cardiac component of the signal was also isolated by applying continuous wavelet decomposition using the Ricker wavelet family at a 5-ms scale.

### Dimensionality reduction

The high-dimensional raw signal data obtained from the 32 channels required further processing before classification modeling. Two strategies were implemented. To begin with, feature extraction was performed, characterizing the peak amplitude (*53*) of each ECAP. A threshold was then determined on the basis of the mean value of the recorded peak amplitudes for each of the 32 recording channels, effectively converting continuous raw signal data into categorical variables.

In the second strategy, the high-dimensional space corresponding to the waveforms extracted after wavelet denoising was reduced using linear and nonlinear dimensionality reduction methods. In particular, principal components analysis (PCA) (*54*, *55*) and uniform manifold approximation and projection (UMAP) (*56*) were applied and compared. UMAP is a nonlinear method that showed to outperform the linear PCA in datasets were nonlinear dependencies were remarkable, although at a higher computational cost.

Dimensionality reduction was necessary as modeling with high-dimensional raw signal data led to overfitting and reduced predictive performance (*57*), which can be solved with feature extraction and dimensionality reduction.

### Machine learning classification modeling

The processed data were used to train the machine learning model, partitioning 60% of the data into a training set and 40% of the data into a classification set. For supervised machine learning, the KNN algorithm (*58*) with automatically optimized hyperparameters (*k* = 1, city block distance metric) was used in MATLAB (2021a). The classified data were aggregated to generate a confusion matrix to calculate overall classification accuracy and classification loss rates for each source of the evoked potential. Tibial versus peroneal SSEP classification were aggregated separately as they were subsets of left/right SSEP. Last, an unsupervised method based on KNN with *k* = 2 (for tibial versus peroneal SSEP dataset) or *k* = 4 (for alternating left/right SSEP/MEP stimulation dataset) was used to identify distinct clusters automatically.

### Acute electronic bypass of SCI

Following implantation of both recording and stimulating I360 devices, control signals for bypass in acute SCI were generated using Intan RHX data acquisition software (Intan Technologies, Los Angeles, CA). By examining action potentials generated around the spinal cord with motor cortex stimulation and confirming their approximate positions on the spinal cord, we referenced the selected channels to reduce ECG and respiratory artifacts to denoise the action potential waveforms. These waveforms can be detected via their rising edges, and their amplitudes were amplified using the native Intan RHX software to generate trigger signals for SCS to reproduce the disrupted hindlimb movement following the acute SCI.

### Hindlimb kinematic capture

The head and trunk of the rat were fixed to the stereotaxic frame, leaving the hindlimbs free for unhindered motion. The video was captured using an 8.0-megapixel camera (IPad Mini 4, Apple Inc., Cupertino, CA), recording at 30 frames/s with 720p high-definition resolution. Deeplabcut version 2.2.0.3, a deep neural network-based pose estimation software, was used to perform marker less pose estimation field (*59*) of the rat hip, knee, and ankle.

### Surgical technique using human cadaveric studies

The devices were tested during a human cadaver session at the Evelyn Surgical Training Centre, Cambridge (UK). Fresh-frozen specimens were used. To achieve the spinal reconstitution with saline, the dura was first exposed by incision above the cervical part of the spinal cord, removal of the spinous process, and full laminectomy at the C4-C6 vertebrae. An incision was made in the dura with an 18-gauge needle, a polyethlye tubing (optical density = 1.4 mm) was inserted into the incision and secured using a purse string suture (Prolene 4-0), and a small amount of cyanoacrylate glue (Locktite) was dispensed around the tube to ensure an adequate seal. The tubing was connected to a flow pump (Flowsteady, model 200). The pump was set to 140 mmHg at a rate of 1.5 liters/min. After 30 s, the dura could be seen to expand; after 60 s, liquid could be seen emerging from the epidural space, indicating either a small leak of the dura or fluid displacement as the dura expanded. The pump was reduced to a pressure of 70 mmHg and 0.5 liters/min. The seal was monitored for at least 30 min before the first implantation to ensure that the seal held and the dura did not leak or expand further.

A midline incision was performed over the thoracic T9 to T10 vertebra, with the dissection taken down to bone. A wide laminectomy was then performed from pedicle to pedicle, followed by a midline durotomy. Sutures were used to retract the dura gently and allow access to the dorso-lateral spinal cord. The dentate ligaments were released to allow the device to slide unopposed around the spinal cord. To introduce the device without impinging on the spinal cord, micro-forceps were used to guide the device laterally along the dura until it reached the ventral aspect of the spinal cord. The device was then advanced until the tip could be seen and retrieved at the contralateral side of the spinal cord.

### Immunohistochemistry

To preserve the morphology of the spinal cord, the tissue was fixed by vascular perfusion with 100 ml of 4% methanol-free paraformaldehyde in PBS with 0.1% sodium azide for 48 hours at 4°C before transfer to 100 ml of 30% sucrose in PBS with 0.1% sodium azide. The tissue is then dissected and mounted in an optimal cutting temperature embedding compound and frozen at −20° to −80°C. Five- to 15-μm-thick tissue sections were prepared using a cryostat (Leica Microsystems, Deerfield, IL) and subsequently thaw-mounted onto gelatine-coated histological slides. The slides are then dried for 30 min on a slide warmer at room temperature.

Glial fibrillary acidic protein (GFAP) was selected as a primary antibody for immunofluorescent staining as it is a marker of astrocyte activity and is up-regulated in neuronal injury (*60*). CD11b is an integrin protein subunit that is used to identify monocytes and microglia (*61*). It was thus selected as a primary antibody to characterize the immune response following SCI and to characterize the foreign body response (*62*). All primary antibodies were obtained from Abcam plc., Cambridge, UK, and secondary antibodies were obtained from Invitrogen, Waltham, MA, USA unless otherwise stated.

For the fluorescent staining of cryostat sections, the slides (LK1) are first rehydrated in wash buffer for 10 min, and the tissue was surrounded with a hydrophobic layer. Nonspecific staining between the primary antibodies and the tissue was blocked by incubating in blocking buffer consisting of 5% donkey serum in PBS for 1 hour at room temperature. Primary antibodies [anti-GFAP antibody (ab7260) used at 1/1000 and anti-CD11b + CD11c antibody (OX42) (ab1211) used at 1/500 in 5% donkey serum in PBS with 0.1% sodium azide] were then applied and left overnight at 4°C.

Subsequently, the tissues were washed three times in 5% donkey serum in PBS with 0.1% sodium azide for 10 min per wash and incubated with secondary antibodies [donkey anti-rabbit immunoglobulin G (IgG) (H + L) highly cross-adsorbed secondary antibody, Alexa Fluor 555 used at 1/500 and donkey anti-mouse IgG (H + L) highly cross-adsorbed secondary antibody, Alexa Fluor 488 used at 1/2000 in 5% donkey serum in PBS with 0.1% sodium azide] for 3 hours at room temperature in the dark before another wash cycle.

The tissues were then incubated in the Vector TrueVIEW Autofluorescence Quenching Kit (Vector Laboratories Inc., Burlingame, CA, USA) for 3 min at room temperature before 4′,6-diamidino-2-phenylindole (1 μg/ml) solution for visualization of cell nuclei and final PBS wash. The slide is then mounted with an antifade media and visualized using a fluorescence microscope (Axioscan.Z1, Carl Zeiss AG, Jena, Germany).

### Circularity index

We used the ImageJ software to calculate the spinal cord circularity index according to their specified formula “4π(area/perimeter^2^).” Measurements were obtained from three distinct sections of each animal’s spinal cord. To mitigate the risk of errors from single measurements and to ensure the accuracy of results, each of these sections was subjected to three separate calculation attempts. We then calculated the mean spinal cord circularity index for each individual animal by averaging the nine individual measurements (three per spinal cord section) taken from each animal. The circularity index has a potential range from 0.0 to 1.0. A value of 1.0 represents a perfect circle, while values nearing 0.0 indicate increasingly elongated polygons.

### Ethics declaration

All in vivo work was carried out in accordance with the UK ASPA 1986 and under the approval of the animal welfare and ethical review body (University of Cambridge).
