# Supplementary material for: Flexible circumferential bioelectronics to enable 360-degree recording and stimulation of the spinal cord
Source: Sci Adv. 2024 May 8;10(19):eadl1230. doi: 10.1126/sciadv.adl1230 (PMC11078185; doi:10.1126/sciadv.adl1230)
Supplement: Supplementary file 1 — Figs. S1 to S11 [file sciadv.adl1230_sm.pdf]

Science Advances
AAAS

Supplementary Materials for

Flexible circumferential bioelectronics to enable 360-degree recording and stimulation of the spinal cord

Ben J. Woodington *et al.*

Corresponding author: Damiano G. Barone, dgb36@cam.ac.uk; George G. Malliaras, gm603@cam.ac.uk

Sci. Adv. **10**, eadl1230 (2024)
DOI: 10.1126/sciadv.adl1230

This PDF file includes:

Figs. S1 to S11

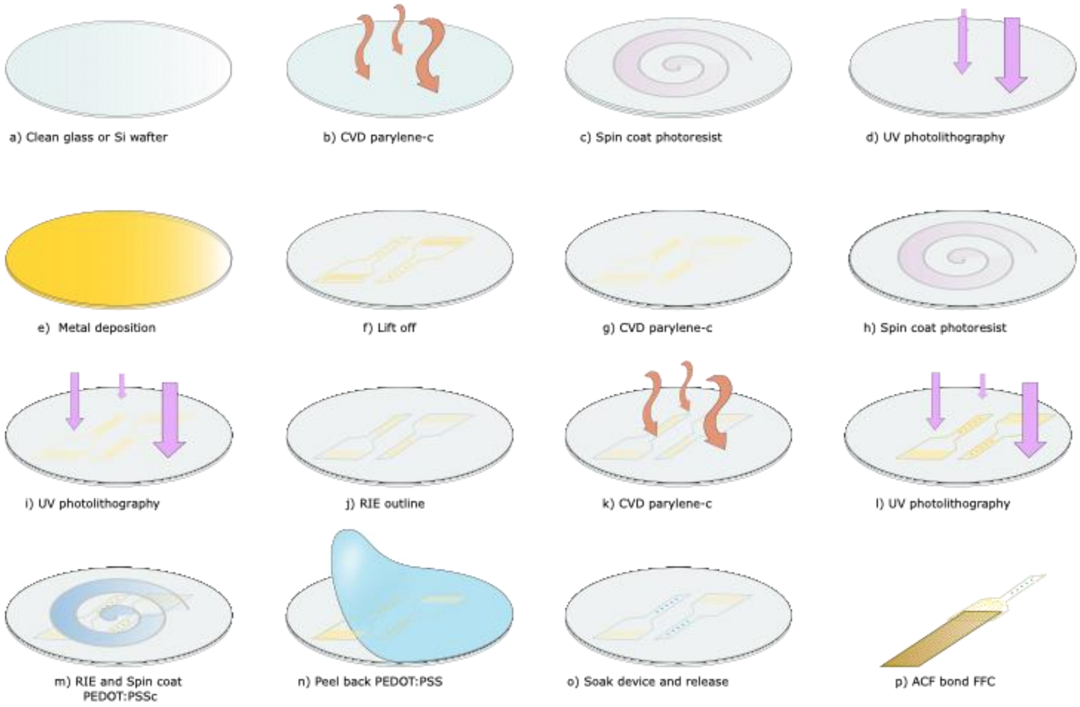

Figure S1 - Fabrication Process: Full fabrication process for the i360 device**.**

In order to verify that the size of the grounded box surrounding the spine model and the spine length does not influence the electric field distribution within the spine, the simulation for a 200 μA injection current was repeated for box sizes of (50 x 50) mm$_2$, (50 x 50) mm$_2$, (100 x 100) mm$_2$, and (200 x 200) mm$_2$ and for a spine length of 5 mm, and 10 mm.

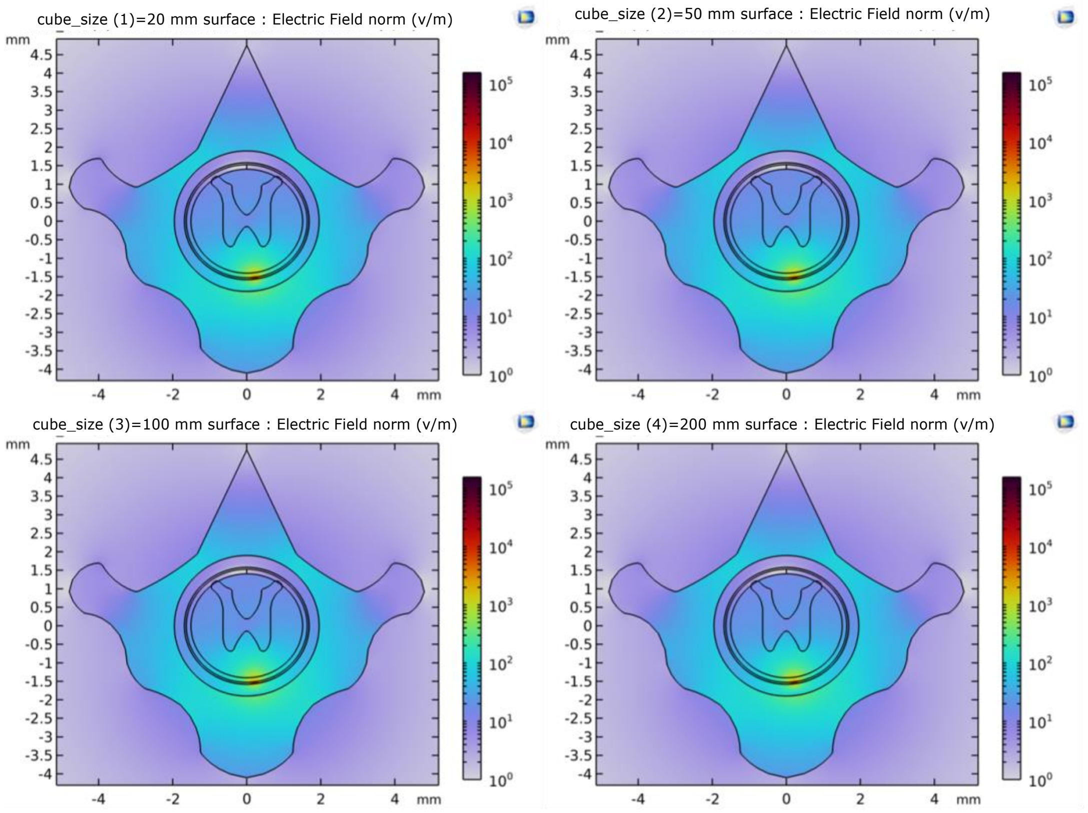

Figure S2 - Stimulation Spread by Grounding Distance: Simulation experiment investigating the effect that global grounding distance has on stimulation spread.

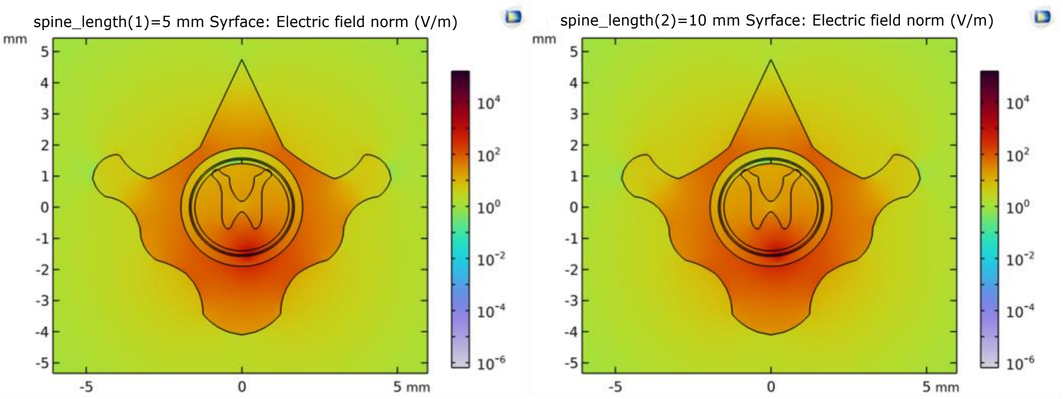

Figure S3 - Stimulation Spread by Cord Length: Simulation experiment investigating the effect that spinal cord length has on stimulation spread.

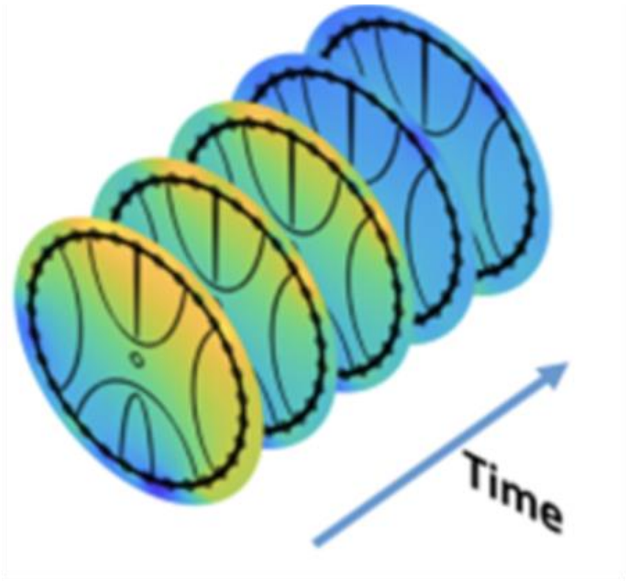

Figure S4 - Neural Recording Evolution: Temporal representation of evolved neural recordings.

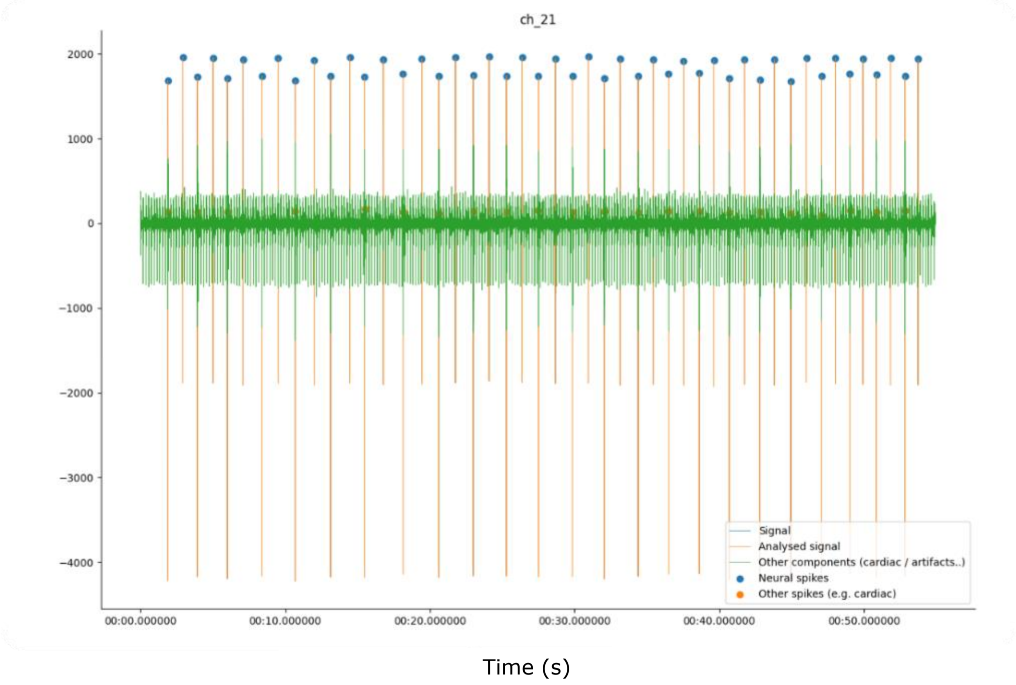

Figure S5 - Wavelet Decomposition Analysis: Wavelet decomposition analysis showing neural signals labelled in blue and other signals labelled in orange. Here other signals are dominated by cardiac peaks.

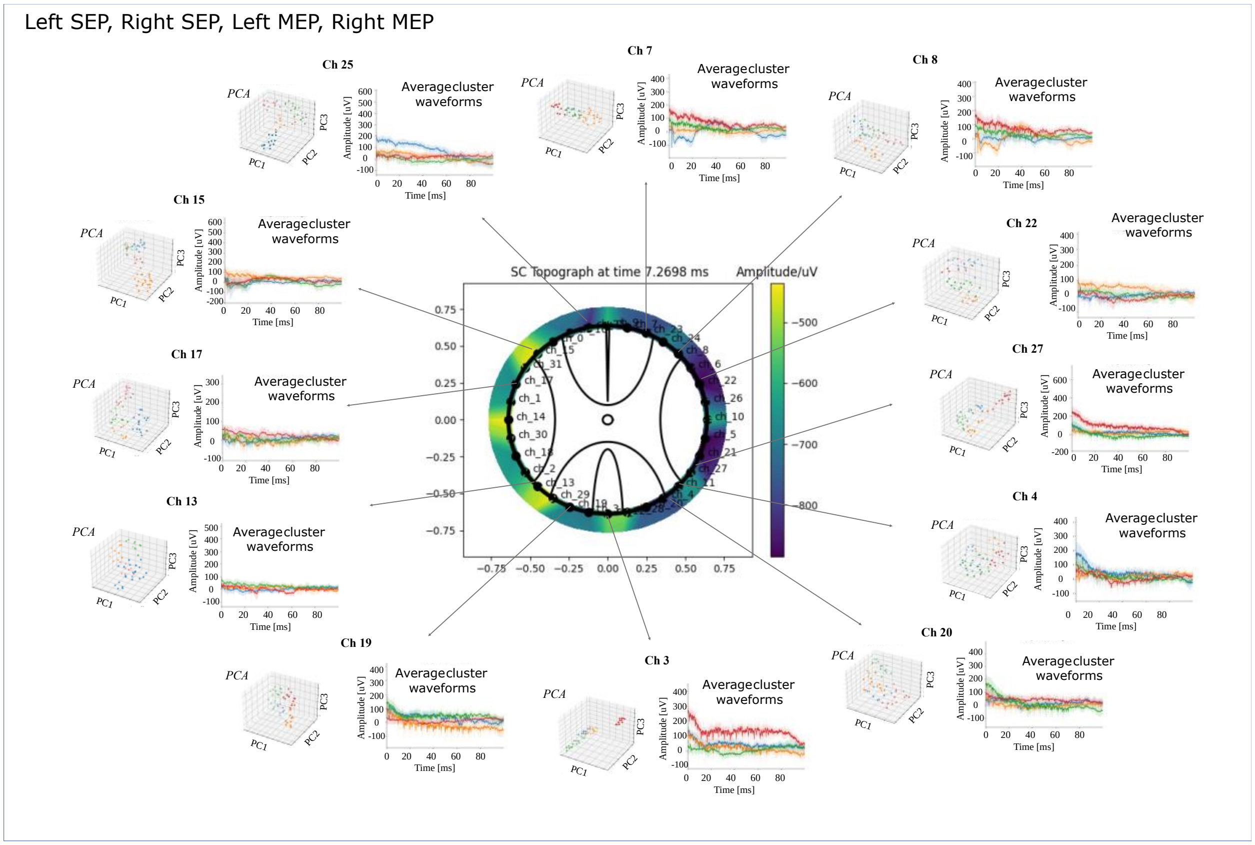

Figure S6 - Principal Component Analysis: Analysis of spinal cord recordings with multiple recordings during an experiment with four inputs: left sensory, right sensory, left motor, and right motor.

Per vs tib

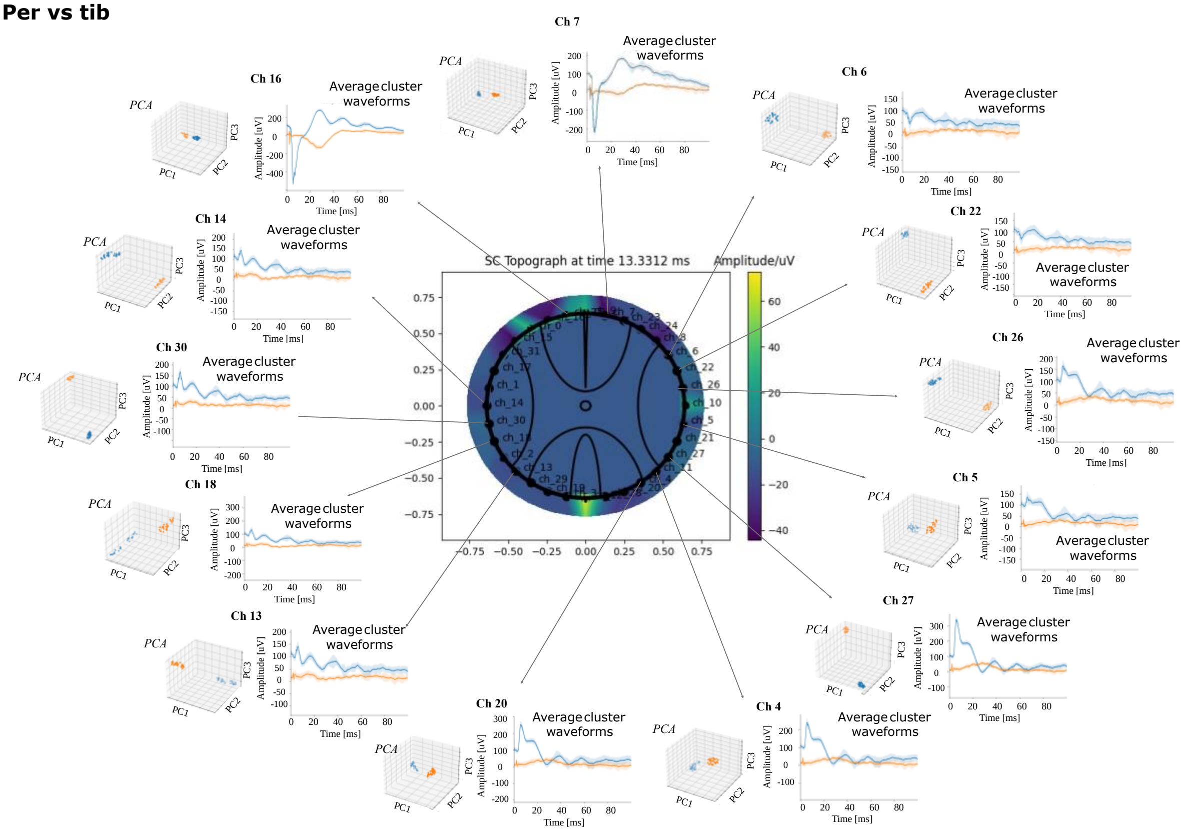

Figure S7 - Principal component Analysis: Analysis of spinal cord recordings with multiple recordings during an experiment with two sensory inputs: Peroneal and Tibial tonic stimulation.

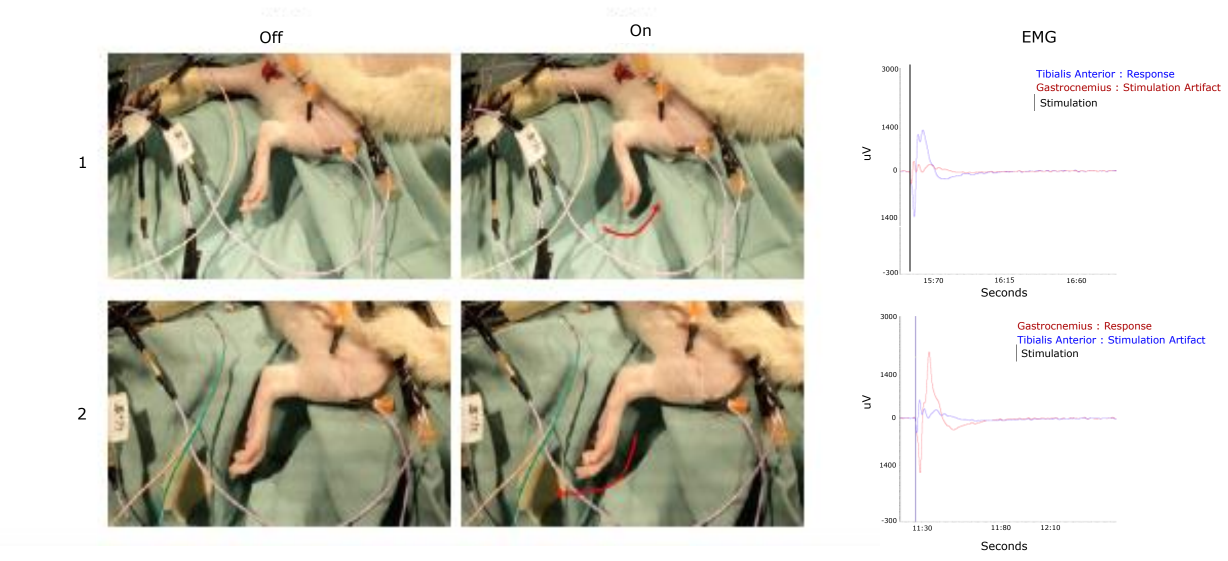

Figure S8 - Muscle-Specific Stimulation: Still captures demonstrating muscle-specific stimulation under electromyography (EMG) monitoring 1. Ankle (Tibialis Anterior) 2. Hamstring (Gastrocnemius). Red arrows show the gross direction of movement.

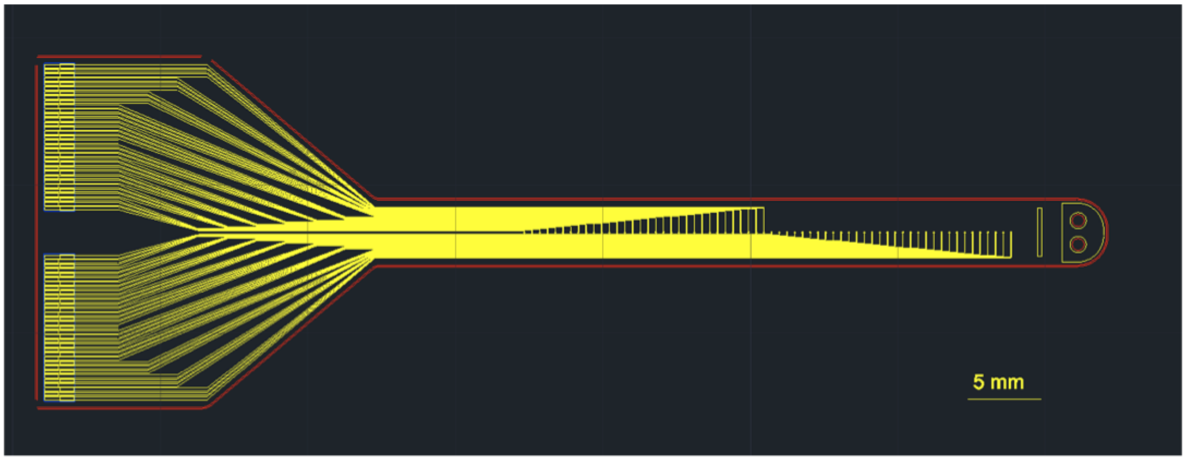

Figure S9 – i360 Device Alteration: Alteration of the i360 device for testing in human cadavers.

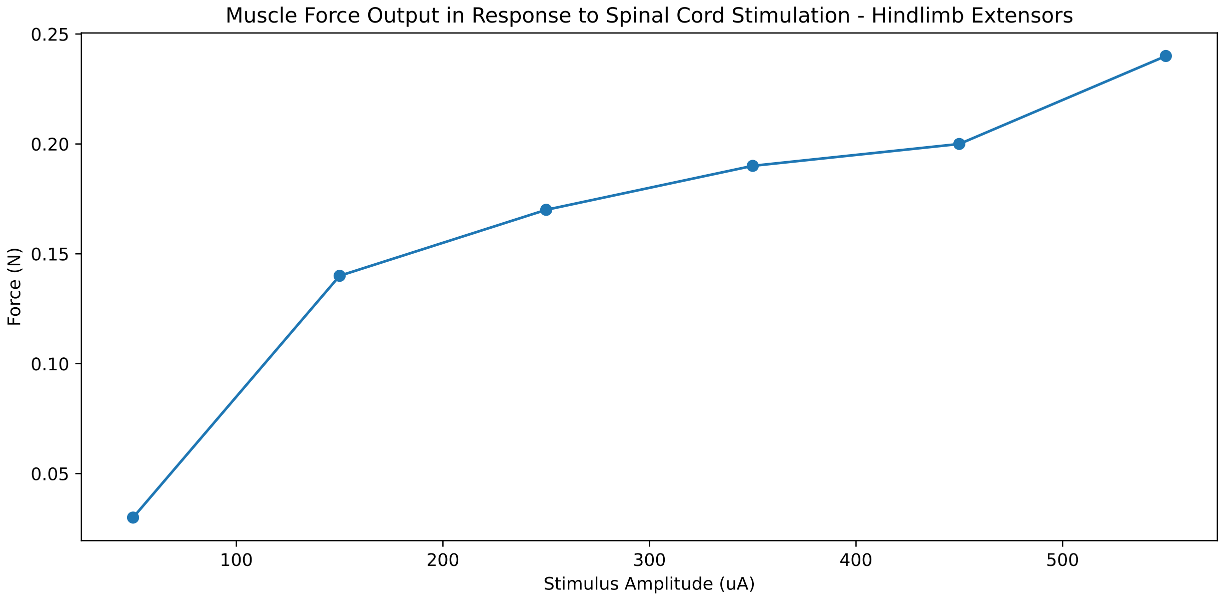

Figure S10 – Hindlimb Extensors Dynamometry : The plot illustrates the force output of hindlimb extensor muscles as a function of stimulus amplitude, from 50 µA to 550 µA.

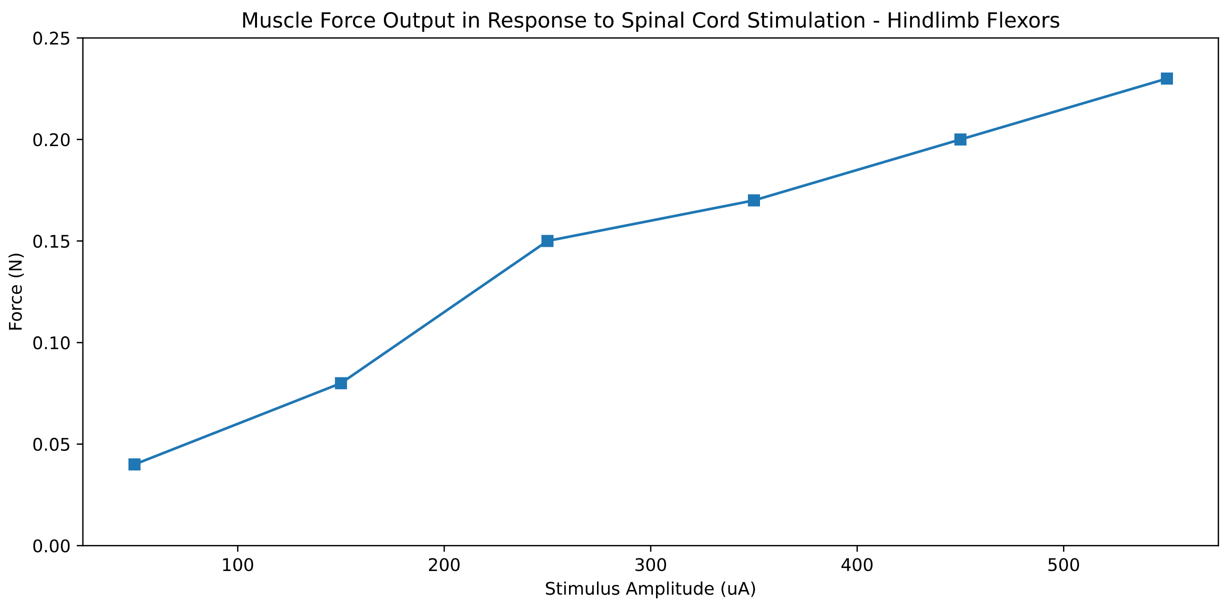

Figure S11 - Hindlimb Flexors Dynamometry : The plot illustrates the force output of hindlimb flexor muscles as a function of stimulus amplitude, from 50 µA to 550 µA.
